# A mutation linked to *degt-1(ok3307)* in *C. elegans* strain VC2633 affects *rpm-1*

**DOI:** 10.17912/micropub.biology.000565

**Published:** 2022-05-05

**Authors:** Eugene Jennifer Jin, Yishi Jin

**Affiliations:** 1 Department of Neurobiology, School of Biological Sciences, University of California San Diego, CA, USA

## Abstract

*C. elegans* strain VC2633 is described to contain the *degt-1(ok3307)* mutation. Here, we report the identification of a loss of function mutation in *rpm-1* that is linked to *degt-1(ok3307)* in VC2633. In homozygous animals of *degt-1(ok3307)* derived from VC2633, we observed neuronal morphology defects that resemble *rpm-1* loss of function. Based on complementation test with *rpm-1* mutants, sanger sequencing of *rpm-1* locus and genome editing, we verified a single nucleotide change designated *rpm-1(ju1928)* in *degt-1(ok3307)* chromosome in VC2633 and derivatives. This mutation alters a conserved Glutamine at amino acid 3089 to Histidine in RPM-1. We generated a new strain of *degt-1(ok3307)* without *ju1928;* the updated genotype of VC2633 strain is *rpm-1(ju1928) degt-1(ok3307).* While currently there are no reported binding partners for the region of RPM-1 containing Glu3089, the neuronal defects associated with the mutant RPM-1 suggest that this region may play roles in regulating RPM-1 activity.

**Figure 1. C. elegans strain VC2633 contains a mutation in rpm-1:**
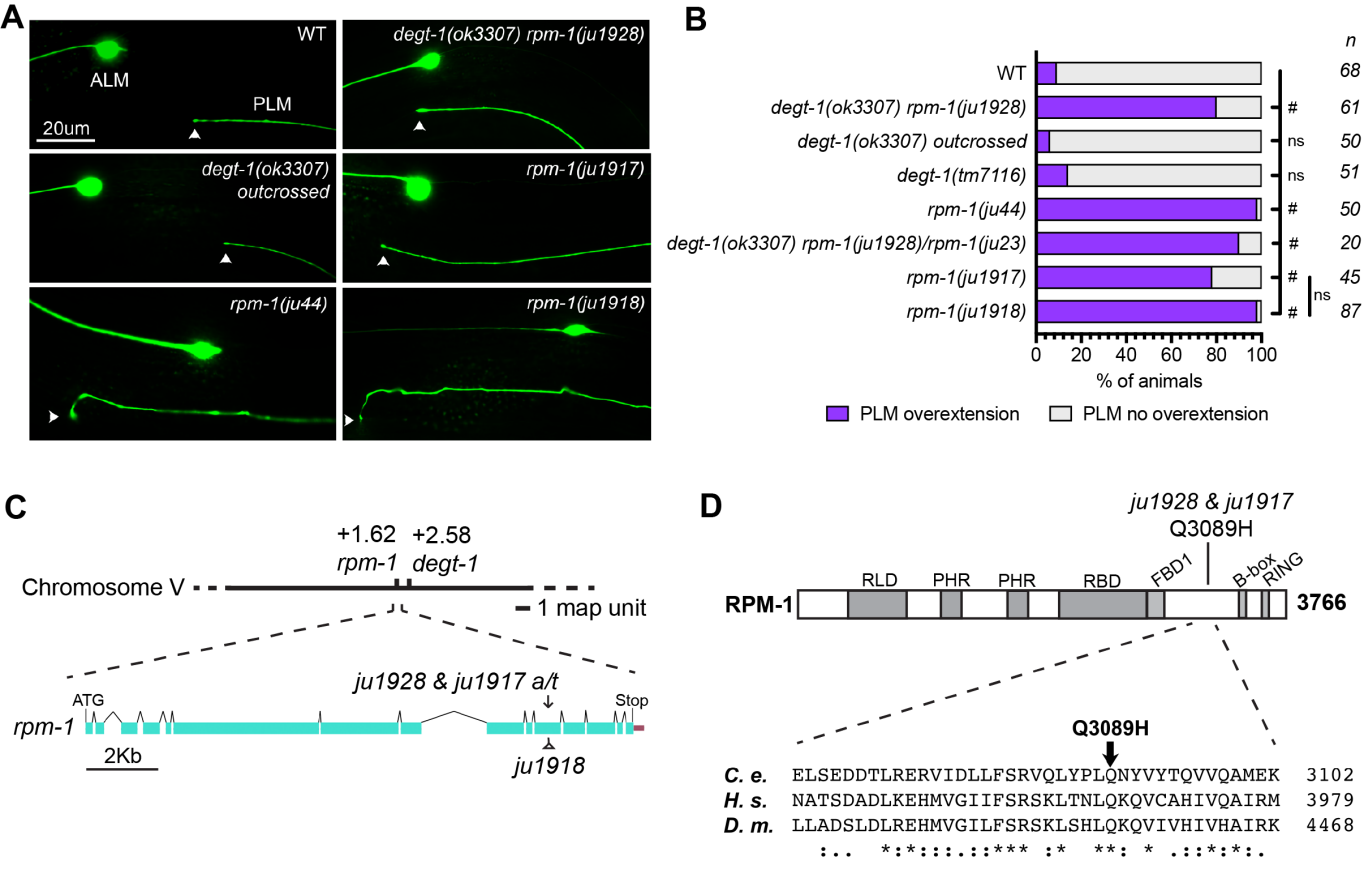
(A) Representative images of PLM axon termination position in relation to ALM soma in L4 animals. ALM and PLM neurons are labelled with *muIs32* . Arrowheads indicate the axon end of PLM. Note that in *rpm-1(ju1928) degt-1(ok3307)* and *rpm-1(ju1917)* mutants, PLM axons terminate shortly past ALM soma, but do not display the hooking phenotype typically observed in *rpm-1(ju44)* and *rpm-1(ju1918)* mutants. (B) Quantification of the % of animals displaying PLM overextension phenotype, defined by at least one of the two PLM axons terminating past the ALM soma. *n* indicates the total number of animals quantified per genotype. Statistics was performed using Chi-squared test followed by Marascuilo procedure. #: significant, ns: not significant. (C) Genomic locus of *rpm-1* and *degt-1,* and gene structure of *rpm-1* with the location of *ju1928* and *ju1917* adenine9267 (in CDS) changed to thymine. *ju1928* is linked to *degt-1(ok3307)* in strain VC2633. *ju1917* was generated by CRISPR/Cas9-mediated genome editing, and contains silent mutations near the *a/t* mutation to change restriction enzyme sites for the ease of genotyping. *ju1918* contains imprecise repair that causes insertion leading to premature stop, resulting in premature stop and truncated protein lacking C-terminal RING domain. (D) RPM-1 protein structure and domains: RCC1-like GEF domain (RLD), Pam, Highwire, RPM-1 family specific domains (PHR), RAE-1 binding domain (RBD), FSN-1 binding domain (FBD1), B-Box Zn finger domains (B-Box) and RING-H2 ubiquitin ligase domain (RING). Below is a multi-species alignment (conserved residues are *) of the Q3089 region for *C. elegans (C. e)* , *H. sapiens (H. s.)* and *D. melanogaster (D. m.)* .

## Description

DEGT-1 is a DEG/ENaC (Degenerin/Epithelial Sodium Channel) protein involved in mechanosensation and is reported to be expressed in many neurons, including the touch neurons and ventral cord motor neurons (Chatzigeorgiou et al., 2010; Tao et al., 2019). The strain VC2633 in CGC has a reported genetic null mutation of *degt-1(ok3307),* generated by the knockout consortium. In the process of examining touch neuron and motor neuron morphology and synapses in *degt-1(ok3307)* mutant, we noticed that the animals exhibit phenotypes that are not present in another allele, *degt-1(tm7116)* , such as overextension of mechanosensory neuron PLM (Fig. 1A-B) and reduced synapse number of GABAergic motor neuron. Moreover, after multiple rounds of outcrossing of VC2633, the abnormal PLM morphology phenotypes remain associated with *degt-1(ok3307),* suggesting that a background mutation is likely linked to *degt-1* on Chromosome V *.*

The PLM overextension phenotype in VC2633 and additional outcrossed strains resembled *rpm-1* , which is 1 cM from *degt-1* on chromosome V (Schaefer et al., 2000; Zhen et al., 2000) (Fig. 1A-C). Complementation test of VC2633 to *rpm-1* mutants shows PLM overextension, supporting that there is a loss of function mutation in *rpm-1* linked to *degt-1(ok3307)* (Fig. 1B). Although VC2633 was derived from UV-TMP mutagenesis, which generally induces genomic deletions, we did not detect major deletions in *rpm-1* by PCR amplification of the locus. We then sanger sequenced multiple regions of *rpm-1* and found a single nucleotide alteration changing Adenine (position 9267 in CDS; gttcaactttatccacttcaAaattacgtgtacactcaagt) to Thymine. This A to T nucleotide transversion results in a missense substitution changing Glutamine3089 to Histidine (Q3089H), designated as *rpm-1(ju1928)* (Fig. 1C-D). We confirmed the presence of *ju1928* in multiple strains that carry *degt-1(ok3307).* We also isolated a strain CZ29001 that contains only *degt-1(ok3307)* (Fig. 1A-B) *,* which will be deposited to CGC.

Gln3089 is a conserved amino acid in an uncharacterized protein region of RPM-1 (Fig. 1D). To further confirm that this mutation disrupts the function of *rpm-1,* we used genome-editing to create a new allele *ju1917* that contains the A/T SNP as well as silent mutations that change nearby restriction enzyme sites for the ease of genotyping. We also obtained an allele *ju1918,* which has imprecise repair that caused insertions leading to a premature stop at 3110th amino acid, and produce an RPM-1 protein lacking the RING domain that is essential for RPM-1’s ubiquitin ligase activity. We observed that both *ju1917* and *ju1918* mutants display PLM overextension (Fig. 1A-B). *ju1917* displays PLM overextension but not the hook phenotype, whereas *ju1918* shows PLM overextension and hooking, indistinguishable from other *rpm-1* null mutations. This analysis further confirms that Q3089H change in strain VC2633 is a partial loss of function mutation in *rpm-1* .

How the Q3089H mutation causes *rpm-1* loss of function remains unclear. Early studies have shown that the region around Q3089H is important for RPM-1 function (Abrams et al., 2008), but is not involved in direct interaction with FSN-1 (F-box Synaptic protein 1) (Sharma et al., 2014). It is possible that the conserved region containing Q3089 may regulate RPM-1’s ubiquitin ligase activity.

## Methods

*Strain maintenance*

All worm strains and crosses were maintained at 20°C on nematode growth media with *Escherichia coli* OP50. L4 animals were used for all analyses in this study.

*Outcrossing*

VC2633 was outcrossed to N2 several times, using PCR-based genotyping for *degt-1(ok3307),* with the following primers: F primer (CGAGATGTACGTTGAGGCAAG), R primer (TTGGAGAATATGACTGGGCCATC) and Internal R primer (GACTTGCGTGGTTATTCTTTCC). Outcrossed *degt-1(ok3307* ) was then crossed into different neuronal reporters, and the phenotypes we report for *rpm-1(ju1928)* were observed in multiple strains, except CZ29001, which we verified by sequencing the absence of *rpm-1(ju1928)* .

*CRISPR/Cas9 genome editing*

To verify the single nucleotide change identified in *rpm-1(ju1928)* , we designed a crRNA for the antisense strand of *rpm-1* : GTGTACACGTAATT **t** TGAAG. The desired nucleotide to be edited (Adenine 9267 in CDS) is within the crRNA and is bolded lower case t in the sequence above. A repair oligonucleotide template for the antisense strand of *rpm-1* with the desired A/T nucleotide change and 4 additional silent mutations was used: GAATAATTTCGCTTCGATTTATCACAAAGCAGCTCGACCTCTTTTTCCATCGCTTGAACAACTTGAGTaTAtA CaTAATT **a** TG AAGaGGATAAAGTTGAACTCTACTGAATAGTAAATCTATAACCCGTTCTCTTAGAGTATCATCTTCGGATAGTTCGC. The A/T SNP position is the bolded lower case, and the 4 silent mutations are lowercase letters. These silent mutations destroy the PAM site (TGG to AGG) and remove cutting sites for restriction enzymes RsaI (GTAC to ATAT), AflIII (ACGTGT to ATGTAT) and BsaAI (TACGTG to TATGTA), and creates a new MslI site (CAAATTACG to CATAATTATG). The new MslI site is underlined in the sequence above.

crRNA (2nmol) and repair template oligonucleotide (ultramer; 4nmol) were ordered from Integrated DNA Technologies (IDT). crRNA was hydrated with duplex buffer for final concentration of 40µM, and repair oligonucleotide template was hydrated with ddH _2_ 0 for working stock concentration of 10µM. Duplex crRNA-tracrRNA was prepared by mixing crRNA and tracrRNA (2µL total volume; 20µM final concentration for both crRNA and tracrRNA), then “melting” the duplex in a thermocycler: 95°C for 30s, 90°C for 30s, 85°C for 30s, ..., 35°C for 30s, 30°C for 30s (Ghanta and Mello, 2020). RNP complex (5µL final volume) was prepared by adding Cas9 protein (IDT; final concentration of 6.1µM) and RNase free water to the 2µL of melted crRNA-tracrRNA duplex (final concentration of 4µM in the RNP complex), then incubated at room temperature for 10 minutes. The following injection mix was prepared: 0.5µM RNP complex, 1µM repair oligonucleotide template, 25ng/mL pRF4 and RNase free water. The injection mix was injected into N2 gonads. F1 roller animals were selected and genotyped by PCR amplification (F primer: CGTAGAGCACCACGATCTGAAG; R primer: CGATAGTGATGAGATTGCTGTCCA), followed by MslI digestion. The desired edits were verified in the final candidates by sanger sequencing with an internal primer: TCAGCGATACTCAACGTGACG.

*Quantification of PLM axon defects*

Defect in PLM mechanosensory neuron’s axon termination was visually scored by determining whether or not the PLM axon terminates past ALM soma. L4 animals were mounted on 10% agarose pads with tiny drops of M9. Zeiss AxioPlan2 microscope with X-Cite 120Q (Excelitas), Plan-Apochromat 63x/1.4 Oil DIC objective and Semrock GFP/DsRed-A-ZHE filter cube (Excitation 468/553; Emission 512/630; Beam splitter 493/574) were used to assess PLM axon termination position relative to ALM soma. Animals with both PLM axons terminating prior to ALM soma were scored “PLM no overextension”. Animals with one or both PLM axon(s) terminating past ALM soma were scored “PLM overextension”.

*Imaging acquisition and processing*

Representative images of ALM and PLM labelled with *muIs32* were acquired using AxioImager M2 with Zeiss HXP 120C, Plan-Apochromat 40x/1.4 Oil DIC (UV) VIS-IR objective, Zeiss filter set 38 HE (Excitation BP 470/40; Emission BP 525/50; Beam splitter FT 495), Zeiss Axiocam 705 monochrome camera, ZEN3.1 pro and 200ms exposure time. Images were acquired as 14bit and saved as czi files. Fiji (Fiji Is Just ImageJ) was used to open the czi files, convert to RGB then save as tif files. Adobe Photoshop was used to compile the image panels and adjust fluorescence intensity levels.

## Reagents

*Strains*

**Table:** 

**Strain**	**Genotype**	**Source**
VC2633	*rpm-1(ju1928) degt-1(ok3307) V*	CGC
FXO7116	*degt-1(tm7116) V*	National Bioresource Project
CF702	*muIs32 [Pmec-7::GFP] II*	(Ch’ng et al., 2003)
CZ29077	*muIs32 [Pmec-7::GFP] II; rpm-1(ju1928) degt-1(ok3307) V*	This study
CZ29001	*muIs32 [Pmec-7::GFP] II; degt-1(ok3307) V*	This study
CZ29002	*muIs32 [Pmec-7::GFP] II; degt-1(tm7116) V*	This study
CZ29004	*muIs32 [Pmec-7::GFP] II; rpm-1(ju44) V*	This study
CZ29244	*muIs32 [Pmec-7::GFP] II; rpm-1(ju1917) V*	This study
CZ29246	*muIs32 [Pmec-7::GFP] II; rpm-1(ju1918) V*	This study
